# Concurrently mapping quantitative trait loci associations from multiple subspecies within hybrid populations

**DOI:** 10.1038/s41437-023-00651-4

**Published:** 2023-10-06

**Authors:** Christie L. Warburton, Roy Costilla, Bailey N. Engle, Stephen S. Moore, Nicholas J. Corbet, Geoffry Fordyce, Michael R. McGowan, Brian M. Burns, Ben J. Hayes

**Affiliations:** 1 Centre for Animal Science, Queensland Alliance for Agriculture and Food Innovation, University of Queensland, St. Lucia, QLD Australia; 2 Agresearch Limited, Ruakura Research Centre, Hamilton, 3214 New Zealand; 3 Formerly Central Queensland University, School of Health, Medical and Applied Sciences, Rockhampton, QLD Australia; 4 The University of Queensland, School of Veterinary Science, St Lucia, QLD Australia; 5 Formerly Department of Agriculture and Fisheries, Rockhampton, QLD Australia

**Keywords:** Genetic variation, Haplotypes, Genomics, Animal breeding

## Abstract

Many of the world’s agriculturally important plant and animal populations consist of hybrids of subspecies. Cattle in tropical and sub-tropical regions for example, originate from two subspecies, *Bos taurus indicus* (*Bos indicus*) and *Bos taurus taurus (Bos taurus)*. Methods to derive the underlying genetic architecture for these two subspecies are essential to develop accurate genomic predictions in these hybrid populations. We propose a novel method to achieve this. First, we use haplotypes to assign SNP alleles to ancestral subspecies of origin in a multi-breed and multi-subspecies population. Then we use a BayesR framework to allow SNP alleles originating from the different subspecies differing effects. Applying this method in a composite population of *B. indicus* and *B. taurus* hybrids, our results show that there are underlying genomic differences between the two subspecies, and these effects are not identified in multi-breed genomic evaluations that do not account for subspecies of origin effects. The method slightly improved the accuracy of genomic prediction. More significantly, by allocating SNP alleles to ancestral subspecies of origin, we were able to identify four SNP with high posterior probabilities of inclusion that have not been previously associated with cattle fertility and were close to genes associated with fertility in other species. These results show that haplotypes can be used to trace subspecies of origin through the genome of this hybrid population and, in conjunction with our novel Bayesian analysis, subspecies SNP allele allocation can be used to increase the accuracy of QTL association mapping in genetically diverse populations.

## Introduction

A number of agriculturally important plant and livestock species are hybrids of genetically diverse subspecies. An example of this hybridisation is that many of the cattle currently in tropical and sub-tropical regions of the world originate from two genetically divergent subspecies, *Bos indicus* and *Bos taurus* (Davis 1993; Bolormaa et al. 2011, 2013). These subspecies diverged between 275,000 (Bradley et al. 1996) and 2 million years ago (Hiendleder et al. 2008). Therefore, it is quite likely that mutations affecting complex traits (quantitative trait loci, QTL) arose independently in the two subspecies. This may be problematic when performing genomic selection and QTL mapping in populations of hybrids and composites of subspecies, as it may result in “ghost” QTL (Kemper et al. 2015a). Ghost QTL are SNP that track QTL in one population, but not in another as the QTL may be absent in the second population (Kemper et al. 2015a). If this is not accounted for, the predicted SNP effect will be inaccurate in the second population. Ghost QTL can decrease the accuracy of both mapping precision and genomic predictions in these multi-breed populations (Kemper et al. 2015a). Therefore, in order to achieve accurate multi-breed and multi-subspecies genomic selection, it will be essential to understand and quantify the effects of differences in genomic architecture between subspecies within genetically diverse populations.

It has been shown that haplotypes may be used to detect regions of the genome that are either *B. indicus* or *B. taurus* in origin (Bolormaa et al. 2011; Koufariotis et al. 2018). Haplotypes are blocks of the genome encompassing SNP alleles that are in close proximity and are likely to be inherited together (Hess et al. 2017). In Australian beef populations, haplotypes have been shown to be able to trace *B. indicus* and *B. taurus* origins in purebred and composite hybrid populations (Bolormaa et al. 2011, 2013; Koufariotis et al. 2018). These studies showed that in Australian populations of *B. indicus* and *B. taurus* cattle, fixed window haplotypes of 9–17 SNP (Bolormaa et al. 2011) or 250 kb (Koufariotis et al. 2018) were sufficient to detect subspecies differences. By assigning subspecies of origin to haplotypes, previous studies have shown subspecies-specific QTL associations in both purebred (Bolormaa et al. 2011; Bolormaa et al. 2013; Koufariotis et al. 2018) and composite (Bolormaa et al. 2011, 2013) tropically adapted beef populations. Bolormaa et al. (2011) also demonstrated that subspecies of origin haplotypes alone could be used to perform genomic selection in a hybrid population using genomic best linear unbiased prediction (GBLUP), however the correlation between estimated breeding value and corrected phenotype for body weight using this method was low (0.08).

In comparison, studies have shown that Bayesian analyses with a prior assumption that SNP can have zero, very small or moderate effects can be used to improve the prediction accuracy of genomic selection in multi-breed populations (Erbe et al. 2012; Kemper et al. 2015b; Rolf et al. 2015). It has been demonstrated that analyses that allow unequal SNP variances often result in improved accuracies in multi-breed genomic selection, particularly for traits that have differing genomic architectures between breeds (Rolf et al. 2015). One such Bayesian analysis that has been shown to be effective in multi-breed populations is BayesR (Erbe et al. 2012; Hayes et al. 2019; Kemper et al. 2015b). BayesR has shown to result in more accurate genomic predictions in multi-breed populations of dairy (Kemper et al. 2015b) and beef cattle (Hayes et al. 2019), especially when the validation population is not highly related to the reference population (Hayes et al. 2019).

There is evidence to suggest that haplotype assigned subspecies of origin can be used to improve the accuracy of QTL association mapping in hybrid populations (Bolormaa et al. 2011). However, there have been no studies investigating the accuracy of QTL association mapping and genomic selection in genetically diverse beef populations using haplotypes to assign subspecies of origin for a Bayesian analysis. In this paper, we propose a novel method of assigning subspecies of origin to SNP alleles using haplotypes to map subspecies-specific genomic architecture in a hybrid population, using a Bayesian analysis. The aims of this research were twofold; (i) to determine the optimal haplotype window to accurately trace subspecies of origin through the genome; and (ii) determine if a model where SNP alleles are assigned to subspecies of origin can be used to improve QTL mapping precision and accuracy of genomic prediction for puberty in a hybrid population of tropically adapted heifers.

## Materials and methods

### Data

#### Reference population

A reference dataset of 1181 purebred animals, genotyped with the Bovine HD array (728,785 SNP, referred to hereafter as 800K), and mapped to reference genome ARS-UCD 1.2 were used for haplotype assignment. This dataset consisted of 868 *B. indicus* animals from a single breed, Brahman, and 313 purebred *B. taurus* animals from five breeds, Angus (*n* = 100), Hereford (*n* = 43), Limousin (*n* = 62), Shorthorn (*n* = 95) and Charolais (*n* = 13). This dataset was used to calculate the haplotype frequency of each haplotype in purebred animals from both subspecies. These haplotype frequencies were consequently used to allocate validation animal haplotypes to a given subspecies.

#### Validation population

Genotypes and puberty phenotypes were sourced from 3695 heifers from three breeds: Brahman (*n* = 979), Santa Gertrudis (*n* = 1802) and Droughtmaster (*n* = 914). Santa Gertrudis and Droughtmaster heifers are stabilised *B. indicus* × *B. taurus* composites, and the Brahman heifers are ‘graded up’ *B. indicus*, consisting of approximately 90% *B. indicus* and 10% *B. taurus* origins (Bolormaa et al. 2011; Koufariotis et al. 2018). Full data recording and phenotype measurement have been described in previous papers (Burns et al. 2016; Engle et al. 2019). Briefly, reproductive maturity score (RMS) is a single ultrasound measurement recorded when a heifer reaches approximately 600 days of age (Burns et al. 2016; Engle et al. 2019). It is measured on a 0–5 scale where 0 = infantile reproductive tract, 1 = small ovarian follicles (<10 mm), 2 = ovarian follicles with a diameter larger than 10 mm, 3 = corpus luteum present, 4 = 10 weeks pregnant, and 5 = > 10 weeks pregnant (Burns et al. 2016; Engle et al. 2019). In previous studies, we have shown that RMS is a moderately heritable trait in this population of heifers, with an estimated heritability of 17% - 35% (Engle et al. 2019; Hayes et al. 2019; Warburton et al. 2020).

All heifers were genotyped with the Geneseek GGP-LD array consisting of 21,121 SNP. These genotypes were imputed up to the BovineHD array of 728,785 SNP (800 K) using FImpute software (Sargolzaei et al. 2014) and a reference dataset of 1500 animals from Brahman, Droughtmaster, Santa Gertrudis, Tropical Composites and other relevant breeds that have been genotyped with the BovineHD array (Hayes et al. 2019).

#### Phasing

Reference alleles were arbitrarily set to ensure the reference allele was the same between reference and validation populations. Genotypes were converted into 0, 1, 2 format where 0 = no copies of alternative allele at a locus, 1 = one alternative allele at a locus and 2 = two copies of the alternative allele at a locus. Phased genotypes were obtained using Eagle version 2.4.1 (Loh et al. 2016) for both the reference and validation datasets.

#### Haplotypes

Haplotypes were generated for both the reference and validation populations using fixed, non-overlapping windows for three haplotype lengths, 50, 100 and 250 kb, using in-house Julia scripts (Bezanson et al. 2017). These scripts take user input of the desired haplotype size and then allocates SNP to a haplotype based upon chromosome position. Each animal has two haplotypes per haplotype window, one paternal and one maternal in origin. The average number of SNP that occur within a haplotype are 13.90, 27.64 and 68.78 for the 50, 100 and 250 kb haplotype windows, respectively (Supplementary Table 1). The average number of SNP assigned to each haplotype window are consistent across chromosomes for each of the haplotype lengths.

A second Julia function was developed to calculate the frequency of each haplotype variant in the reference population, within a haplotype window, for each subspecies. Within each window there may be up to 2^*m*^ variants, where *m* is the number of SNP that fall within the haplotype window (Bolormaa et al. 2011). To calculate the frequency of the occurrence of each haplotype variant in each subspecies, our function calculates the number of unique haplotype variants that occur within each window. Haplotype frequencies were used to estimate the subspecies of origin for each haplotype in the validation population, as described below.

### Subspecies of origin calculations

Subspecies of origin was calculated using a method derived from Bolormaa et al. (2011). In this method, each haplotype was assigned a *b*-value, which is the probability that a haplotype variant was *B. indicus* in origin (Bolormaa et al. 2011) (Eq. 1).

1 $$b=\frac{{{pBi}}_{j}}{{{pBi}}_{j}+{{pBt}}_{j}}$$

Where *pBi* is the frequency of the *j*th haplotype in the *B. indicus* reference animals and *pBt* is the frequency of the *j*th haplotype in the *B. taurus* reference animals. Each of the *m* SNP within a haplotype were allocated to either the *B. indicus* or *B. taurus* origin based upon haplotype *b* estimates. Haplotypes were considered to be *B. indicus* in origin if *b* ≥ 0.5 and *B. taurus* in origin if *b* < 0.5. In the instance that a haplotype within the validation population did not occur within either of the reference subspecies, Hamming distance methods were used to calculate the probability of the haplotype belonging to each of the two subspecies (Van der Loo 2014; Gomez 2015). The Hamming distances were summed to obtain the sum of the *B. indicus* Hamming distances, sum(Bi), and the sum of the *B. taurus* Hamming distances, sum(Bt), respectively. The probability of the undefined haplotype belonging to either subspecies was calculated using Eqs. (2) and (3). The undefined haplotype was henceforth allocated to the subspecies of origin with the largest probability calculation; *B. indicus* (Prob(Bi)) or *B. taurus* (Prob(Bt)).

2 $${\rm{Prob}}\left({\rm{Bi}}\right)=\frac{{\rm{sum}}({\rm{Bi}})}{{\rm{sum}}\left({\rm{Bi}}\right)+{\rm{sum}}({\rm{Bt}})}$$

3 $${\rm{Prob}}\left({\rm{Bt}}\right)=\frac{{\rm{sum}}({\rm{Bt}})}{{\rm{sum}}\left({\rm{Bi}}\right)+{\rm{sum}}({\rm{Bt}})}$$

As each animal has two haplotypes per window, *b*-values were used to estimate if an animal was homozygous *B. indicus* (Bi), homozygous *B. taurus* (Bt) or *B. indicus × B. taurus* (Bx) hybrid for each haplotype window. Each of the *m* SNP within the haplotype window was assigned this subspecies of origin (Bi, Bt or Bx). We then used this classification of subspecies of origin when building the X-matrix for Bayesian analysis.

### Breed subspecies content

After assigning *b*-values to each haplotype for each of the validation heifers, we calculated the average *B. indicus* percentage of each of the validation heifers, based upon haplotype subspecies of origin. This data was then used to calculate the average *B. indicus* percentage of each of the three validation breeds, Brahman, Santa Gertrudis and Droughtmaster. These averages were compared to the original theoretical breed compositions from these hybrid breeds to determine if haplotypes were able to track *B. indicus* content through the genome of these populations.

### X-matrix

**Fig. 1:**
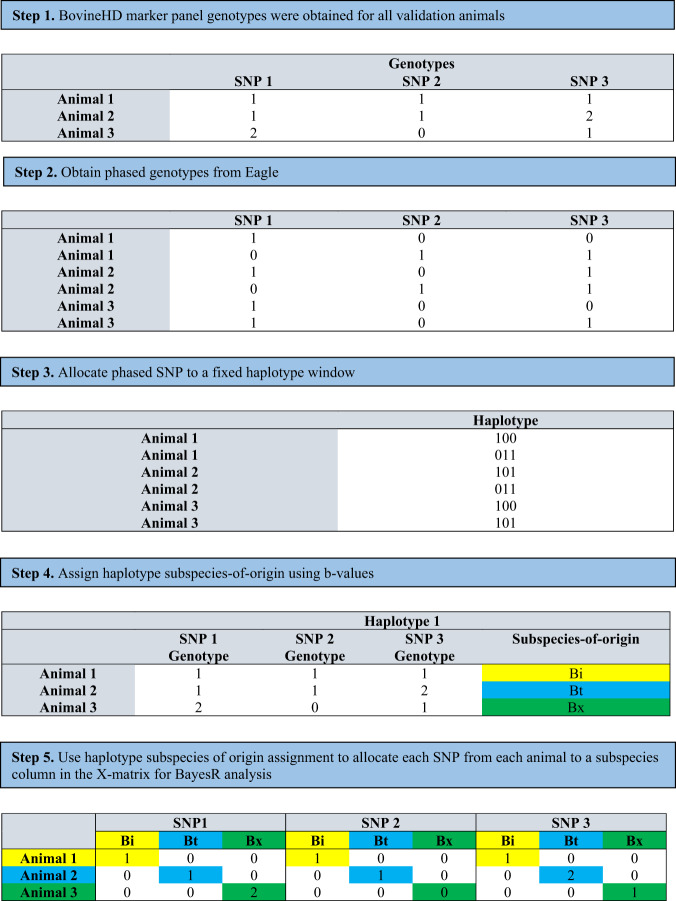
Example showing the process of using haplotype defined subspecies of origin to assign SNP to a subspecies-specific X-matrix for BayesR analysis. As each animal has two haplotypes per window, subspecies of origin is assigned using the combined subspecies assignment from both haplotypes, where Bi is homozygous Bos indicus origin, Bt is homozygous Bos taurus origin and Bx is heterozygous Bos indicus x Bos taurus origins.

To account for genomic architecture in genetically divergent populations, a customised X-matrix was designed to allow simultaneous estimation of multiple subspecies-specific effects in a BayesR analyses. This X-matrix can be used to estimate the effect of an animal being homozygous *B. indicus*, homozygous *B. taurus* or the effect of being a composite *B. indicus × B. taurus* (capturing heterosis) at each SNP, simultaneously. The X-matrix has the dimensions of *nanim × 3nsnp* where *nanim* is the number of animals in the validation population, and *nsnp* is the number of SNP on the marker panel that were used to construct haplotypes. Within the X-matrix, each SNP is represented by three columns, one for homozygous *B. indicus* (Bi), one for homozygous *B. taurus* (Bt) and one for *B. indicus × B. taurus* (Bx) (Fig. 1). Phased genotype data (0, 1 or 2) was used to populate the matrix, with subspecies of origin (Bi, Bt, Bx) being used to allocate the phased genotype information to the appropriate X-matrix column for each SNP and animal (Fig. 1). For example, if an animal has been classified as being homozygous *B. indicus* for a SNP, that SNP will have the count of the alternative allele (0 or 2 from the phased genotypes) added to the Bi column in the X-matrix, and both the Bt column and the Bx column will be empty (0). The resulting X-matrix fits all SNP from the BovineHD array, assigned to an estimated subspecies of origin, that was calculated from the fixed window haplotype, using the reference population haplotype frequencies.

### BayesR models

BayesR analyses used the model:

4 $${\bf{RMS}}={\bf{1}}{\bf{n}}{\rm{\mu }}+{\bf{cg}}+{\bf{age}}+{\bf{Xg}}+{\bf{e}},$$

where **RMS** is a vector of phenotypes, **1n** is a vector of ones, µ is the overall mean, **cg** is contemporary group defined as year, herd and season and **age** is age at measurement fitted as a covariate. There were no mixed breed contemporary groups in this dataset therefore, breed was not fitted as a covariate in the model as contemporary group described all of the variation due to breed in this analysis. **X** is the customised X-matrix described above with dimension *nanim x 3nsnp*, **g** is a vector of SNP effects with a distribution g$\sim N({\bf{0}},{{\rm{\sigma }}}_{{\rm{i}}}^{2})$. The parameter ${{\rm{\sigma }}}_{{\rm{i}}}^{2}$ is one of four distributions: ${{\rm{\sigma }}}_{{\rm{i}}}^{2}$ = {0, 0.0001, 0.001, or 0.01}$\times {{\rm{\sigma }}}_{{\rm{g}}}^{2}$, for the ith SNP distribution and ${{\rm{\sigma }}}_{{\rm{g}}}^{2}$ is the estimated genetic variance of the trait (Erbe et al. 2012). Erbe et al. (2012) described two latent parameters for BayesR, the first parameter, ${\rm{b}}({\rm{i}},{\rm{k}})$ defines whether the estimated SNP effects follow a normal distribution and ${\rm{k}}=(1,2,3,4)$:

5 $${p}\left({{g}}_{{i}}|{b}\left({i},{k}\right)\right)=\left\{\begin{array}{l}0,\\ \frac{1}{\sqrt 2{{\pi }}{{{\sigma }}}_{{i}}^{2}[{k}]}\exp \frac{{{g}}_{{i}}^{2}}{2{{{\sigma }}}_{{i}}^{2}[{k}]}\end{array}\begin{array}{l}{\rm{b}}\left({i},1\right)=1\\ {b}\left({i},{k}\right)=1({k}=2,3,4)\end{array}\right.$$

The proportion of SNP that fall into each of the four distributions are defined by the parameter **Pr** where the prior of ${\bf{\Pr }}$ is sampled from a Dirchlet distribution, ${\bf{\Pr }}{\boldsymbol{ \sim }}{\bf{Dirchlet}}{\boldsymbol{(}}{\boldsymbol{\alpha }}{\boldsymbol{)}}$, and ${\boldsymbol{\alpha }}{\boldsymbol{=}}\left[{\boldsymbol{1}}{\boldsymbol{,}}{\boldsymbol{1}}{\boldsymbol{,}}{\boldsymbol{1}}{\boldsymbol{,}}{\boldsymbol{1}}\right]$ (Erbe et al. 2012). Furthermore, the probability that SNP i falls into each distribution can be defined as:

6 $$\begin{array}{l}{p}\left({{g}}_{{i}}|{{\Pr }}\right)={\Pr }_{1}\times N\left(0,0\times {{{\sigma }}}_{{g}}^{2}\right)+{\Pr }_{2}\times {N}\left(0,0.0001\times {{{\sigma }}}_{{g}}^{2}\right)\\\qquad\qquad\qquad+\,{\Pr }_{3}\times {N}\left(0,0.001\times {{{\sigma }}}_{6}^{2}\right)+{\Pr }_{4}\times {N}(0,0.01\times {{{\sigma }}}_{{g}}^{2}).\end{array}$$

BayesR analyses were conducted with Gibbs sampling using 50,000 iterations, discarding the first 20,000 as burn-in (Moser et al. 2015). BayesR analyses are able to simultaneously estimate SNP effects and genomic EBV (GEBV) of selection candidates (Moser et al. 2015) using the equation:

7 $${\rm{GEBV}}={\bf{X}}{\hat{{\bf{g}}}}.$$

We compared results from this model to a control BayesR analysis, not modelling different origins of QTL alleles. The X-matrix for this control analysis was formed from the 800 K marker panel genotypes with no subspecies of origin adjustments, and has the dimensions *nanim × nsnp*.

### Genomic selection

Genomic selection was performed in the validation population animals using both control and subspecies-specific BayesR analyses in a fivefold cross-validation method. All the validation population heifers were randomly split into five, even sized, mixed breed groups and the analysis was repeated five times using 80% (*n* = 2956) as training and the remaining 20% (*n* = 739) as validation, each time. This strategy of allocating validation group was designed to reflect the mixed breed, mixed subspecies populations in the north Australian beef industry and was used to test the efficacy of our model at accurately predicting GEBV in these mixed breed cohorts. Each animal only occurred in a single validation group and the validation groups remained the same for each of our analyses.

Prediction accuracy was calculated as the correlation of the GEBV and the phenotype adjusted for both age at measurement and contemporary group effects, divided by the square root of the heritability of the RMS trait. We have previously estimated the heritability of RMS to be 0.20 (Warburton et al. 2020) and have chosen to continue to use this heritability estimate for consistency. Standard errors were calculated as the standard error of the mean of the prediction accuracy estimates of the five validation groups. Similarly, bias was calculated for the five validation groups as the regression of GEBV on adjusted phenotype.

### QTL association mapping

In addition to genomic selection, BayesR was also used to conduct QTL association mapping of subspecies-specific SNP in the validation population heifers. This analysis used the model in Eq. (4) to estimate subspecies-specific SNP effects and posterior probabilities using all animals in the validation population. The design of our custom X-matrix allowed the three subspecies of origin effects to be estimated simultaneously in the BayesR analysis. This resulted in estimated SNP effects and posterior probabilities for three subspecies for each SNP, *B. indicus* (Bi), *B. taurus* (Bt) and *B. indicus* × *B. taurus* (Bx). Similar to the genomic selection analysis, a *multi-breed* control analysis was also performed where no subspecies-specific effects were assumed. In addition, for comparison purposes, we also performed a genome wide association study (GWAS) analysis in GCTA (Yang et al. 2011) using the subspecies-specific X matrix SNP. This analysis was performed to determine if the BayesR analysis and GCTA GWAS analyses identified the same subspecies-specific SNP influencing RMS in this population of heifers (Supplementary Fig. 1).

After performing Bayesian analyses, estimated SNP effects and posterior probabilities of inclusion were plotted for the control analysis and each of the three SNP origins, simultaneously, using the R package CMplot (Yin et al. 2021). Posterior probability of inclusion of a SNP is calculated using the equation:

8 $${{\rm{PIP}}}_{{\rm{Inclusion}}}={{\rm{PIP}}}_{2}+{{\rm{PIP}}}_{3}+{{\rm{PIP}}}_{4}$$

Where PIP_2_, PIP_3_, and PIP_4_ are the posterior probabilities of a SNP falling into distributions 2, 3 or 4 in the BayesR model. Alternatively, PIP_Inclusion_ can be calculated as the probability of a SNP not having a zero effect (PIP_Inclusion_ = 1 − PIP_1_).

## Results

### Optimal haplotype size

The Brahman breed was originally developed in the United States of America after a number of *B. indicus* breeds were imported from India, the Ongole, Krishna, Gujarat and Gir (Koufariotis et al. 2018). During the Brahman breed formation these tropically adapted breeds were crossed with the local *B. taurus* breeds, a process referred to as ‘grading up’ the *B. taurus* breed to *B. indicus* (Briggs and Briggs 1980; Koufariotis et al. 2018). Studies have shown that due to the process of ‘grading up’ during the breed formation (Briggs and Briggs 1980), the Australian Brahman genome is approximately 10% *B. taurus* in origin (Bolormaa et al. 2011; Koufariotis et al. 2018), so we have assumed the theoretical *B. indicus* percentage of these heifers is 90%. Both the Droughtmaster and Santa Gertrudis breeds are composite *B. indicus* × *B. taurus* breeds, the Droughtmaster is approximately 50% *B. indicus* (The Droughtmaster Society Australia, n.d) and the Santa Gertrudis is approximately 37% *B. indicus* in origin (Mallett 1959).

Comparison of the average *B. indicus* percentage in Table 1 shows that both the 50 kb and 100 kb haplotype windows have similar average *B. indicus* percentage to the theoretical *B. indicus* percentage from the original breed compositions. In comparison, the 250 kb window appears to consistently overestimate the *B. indicus* percentage in each of the three breeds, when compared to the theoretical *B. indicus* percentage. However, observation of the standard deviations of these estimates show that the estimated *B. indicus* percentage of the 250 kb haplotype window is not significantly different from the theoretical breed percentage.

**Table 1:** Theoretical *Bos indicus* percentage (%) from known breed origins and minimum (Min), maximum (Max), mean (*μ*) and standard deviation (sd) of estimated *Bos indicus* percentage (%) of heifers within each of the three breeds across each of the haplotype window sizes (50, 100 and 250 kb).

	Theoretical	50 kb				100 kb				250 kb			
	*μ*	Min	Max	*μ*	sd	Min	Max	*μ*	sd	Min	Max	*μ*	sd
Brahman	90	46	89	84	0.05	49	94	89	0.05	57	98	94	0.04
Droughtmaster	50	31	65	50	0.05	35	69	54	0.05	47	77	63	0.04
Santa Gertrudis	37	27	48	36	0.02	30	52	39	0.02	41	60	49	0.02

**Fig. 2:**
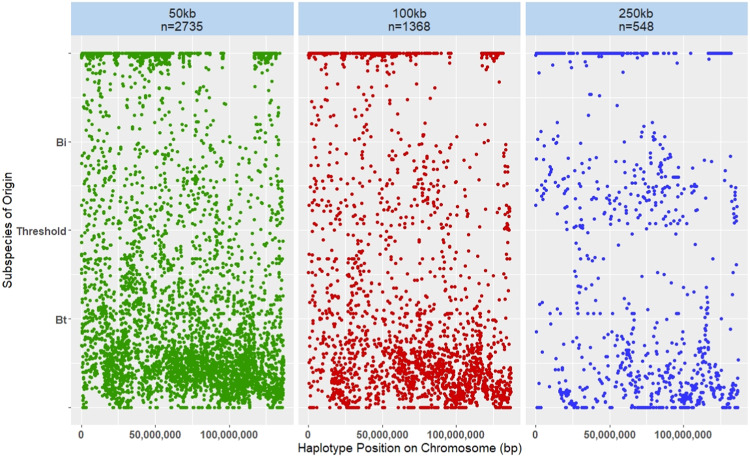
Distribution of *b-*values for each haplotype within the three haplotype windows, 50, 100 and 250 kb, for a single Droughtmaster heifer on Chromosome 2. *X*-axes show chromosomal position and *y*-axes show the distribution of *b*-values, with *b*-values between 0.5 and 1 indicating *Bos indicus* (Bi) origins and *b*-values less than 0.5 indicating *Bos taurus* (Bt) origins.

To further investigate the effect of haplotype length on subspecies of origin assignment, we plotted the *b*-values of a single Droughtmaster heifer from Chromosome 2, to observe the difference in *b*-value distribution between the haplotype windows (Fig. 2). Chromosome 2 was arbitrarily selected and plotted, and the distribution of *b*-values was consistent across chromosomes (results not shown). As the size of the haplotype windows increases, the number of haplotype segments across the chromosome decreases, *n* = 2735, 1368 and 548 for the 50 kb, 100 kb and 250 kb haplotype windows, respectively. However, there is a more consistent distribution of *b*-values across the continuum for the 50 and 100 kb than the 250 kb haplotype windows. The 250 kb haplotype windows show more clustering of *b-*values around the 0.5 threshold and towards the *B. taurus* axis.

### Genomic selection

Accuracy of genomic prediction was not significantly improved when using a subspecies-specific X-matrix, however, the 100 kb haplotype window defined X-matrix resulted in the highest prediction accuracy for RMS in this population of heifers (Table 2). Furthermore, there was a marked improvement in estimation bias using the subspecies-specific X-matrix in genomic predictions, particularly when using subspecies allocations defined with the 100 and 250 kb haplotype windows.

**Table 2:** Prediction accuracy and bias estimates from genomic prediction analyses in BayesR using a multi-breed control and subspecies assigned SNP on the BovineHD array, with subspecies of origin being determined in the 50, 100 and 250 kb haplotype windows with standard errors in parentheses.

	Prediction accuracy	Bias
Control	0.43 (0.05)	0.88 (0.10)
50 kb	0.44 (0.05)	0.86 (0.12)
100 kb	0.45 (0.04)	1.00 (0.10)
250 kb	0.43 (0.03)	1.02 (0.09)

One of the aims of this study was to determine the optimal haplotype window length to estimate subspecies-specific effects in this hybrid population. Based upon the ability to track *B. indicus* content through the genome and the improvements in both prediction accuracy and bias in genomic predictions, the 100 kb haplotype window appears to be the optimal haplotype length in this population. As such, we used the X-matrix created using the 100 kb haplotype windows for QTL association mapping to further define subspecies differences in genetic architecture in this population of heifers.

### QTL association mapping

**Fig. 3:**
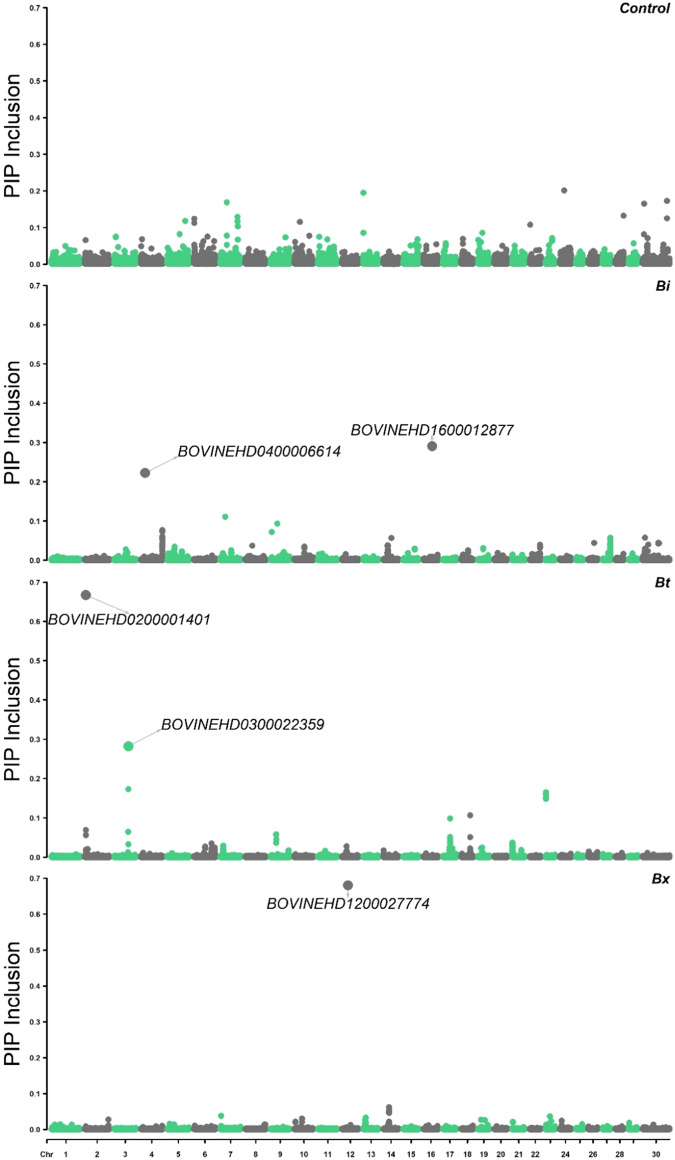
Posterior probability of inclusion (PIP Inclusion) of multi-breed control SNP (Control) and of subspecies-specific SNP effects, *Bos indicus* (Bi), *Bos taurus* (Bt) and *Bos indicus* × *Bos taurus* (Bx), using the 100 kb haplotype-defined X-matrix from BayesR analysis. *X*-axes show chromosomal position and *y*-axes show posterior probability of inclusion for each SNP. The first panel shows the multi-breed control analysis (Control) SNP followed by the *Bos indicus* (Bi) SNP, *Bos taurus* (Bt) SNP and *Bos indicus* × *Bos taurus* (Bx) SNP posterior probabilities.

Similar to the genomic selection analysis, QTL association mapping was performed using both the subspecies-specific X-matrices defined by different haplotype windows, and a multi-breed control analysis where the X-matrix does not contain subspecies of origin effects. This multi-breed control analysis was unable to identify some subspecies-specific SNP of moderate effect and frequency that have an effect on the RMS trait in this population of heifers (Fig. 3). The maximum posterior probability of inclusion of any SNP in the control analysis was 0.20. Whereas, in the subspecies-specific analysis, there were five subspecies-specific SNP that had posterior probabilities of inclusion greater than 0.20 (annotated in Fig. 3).

**Fig. 4:**
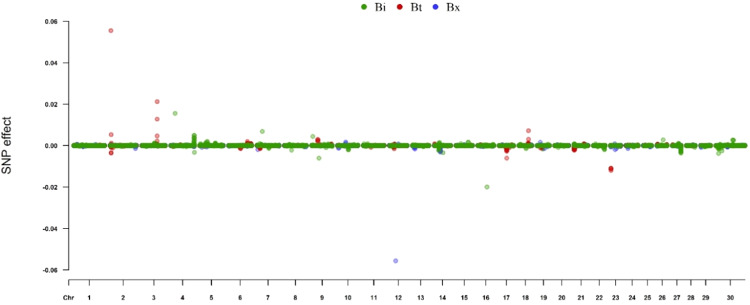
Estimated subspecies-specific SNP effects, *Bos indicus* (Bi), *Bos taurus* (Bt) and *Bos indicus* x *Bos taurus* (Bx), from BayesR analysis using the 100 kb haplotype window-defined subspecies-specific X-matrix. *X*-axes show chromosomal position and *y*-axes show estimated SNP effect. *Bos indicus* SNP are shown in green, *Bos taurus* SNP are in red and *Bos indicus* x *Bos taurus* SNP are in blue.

These results show that there are subspecies-specific effects for RMS, with some SNP having a moderate effect upon the trait (Fig. 4). In particular, there are *B. taurus* (Bt) and *B. indicus* × *B. taurus* (Bx) specific SNP that appear to have a moderate effect upon RMS in this population of heifers, and these SNP do not appear to have the same magnitude of effect in the other subspecies.

There were five SNP in particular that had moderate to large posterior probabilities of inclusion in the subspecies-specific BayesR analysis. Two SNP were identified from *B. indicus* (Bi) origins, BOVINEHD0400006614 and BOVINEHD1600012877, two were identified from *B. taurus* (Bt) origins, BOVINEHD0200001401 and BOVINEHD030022359, and one was identified from *B. indicus × B. taurus* (Bx), BOVINEHD1200027774. Of these five SNP, four were situated in protein-coding regions of genes (Table 3).

**Table 3:** The SNP, subspecies of origin (Subspecies) chromosomal position (Chr:Pos), control analysis allele frequency (Control Freq), *Bos indicus* allele frequency (Bi Freq), *Bos taurus* allele frequency (Bt Freq), Bos indicus x Bos taurus allele frequency (Bx freq), genes and biological function associated with the four subspecies-specific SNP identified using the subspecies-specific X-matrix in BayesR analysis.

SNP	Subspecies	Chr:Pos	Control Freq	Bi Freq	Bt Freq	Bx Freq	Gene	Biological Function	Identified Species
BOVINEHD1200027774	Bx	12:31772526	0.98	0.99	1.00	0.99	*FLT1*	Follicle growth, female fertility	Sheep^a,b^ Rats^c^
BOVINEHD0200001401	Bt	2:4981764	0.87	0.98	0.95	0.94	*MY07B*	Pre-eclampsia	Humans^d^
BOVINEHD0300022359	Bt	3:771374444	0.64	0.98	0.76	0.89	*WLS*	Ovulation and female fertility	Mice^e^
BOVINEHD0400006614	Bi	4:22464663	0.20	0.82	0.67	0.71	*DGKβ*	Dopaminergic transmission in pituitary gland	Rats^f^

^a^Xu et al. 2018.

^b^Ghiasi and Abdollahi-Arpanahi 2021.

^c^Celik-Ozenci et al. 2003.

^d^Mohamad et al. 2020.

^e^Chen et al. 2021.

^f^Hozumi et al. 2010.

## Discussion

To our knowledge, this is the first instance where BayesR has been used to perform QTL association mapping from multiple subspecies in a hybrid population. This novel approach of assigning subspecies of origin to SNP and using a customised Bayesian analysis has allowed us to quantify the effect of differing genomic architecture for RMS in a hybrid population of cattle. Our results show that there are differences in SNP effects between each of the subspecies, and these differences are not being reflected in the multi-breed control analysis of the same population of heifers. Furthermore, our results have also demonstrated that there are some SNP that show interactions when inherited from both subspecies, most notably BOVINEHD1200027774. This SNP had minimal effect upon RMS in homozygous *B. indicus* or homozygous *B. taurus* heifers, however, it had an increased effect in heifers that were heterozygous *B. indicus × B. taurus* at this locus. One of the main advantages of our method is the ability to detect both subspecies-specific effects, and any interactions that may occur at hybrid loci simultaneously, to more accurately understand the underlying genomic architecture of diverse populations.

Studies in other species have shown that using breed of origin allele assignment can increase the prediction accuracy in simulated (Ibánez-Escriche et al. 2009) and pig populations (Sevillano et al. 2019). However, the magnitude of this improvement in prediction accuracy has been low (Ibánez-Escriche et al. 2009; Sevillano et al. 2019). Similarly, our study has shown that use of subspecies of origin assignment to alleles has resulted in small, but not significant improvements in prediction accuracy for reproductive maturity score, a lowly heritable trait (*h*^2^ = 0.20). Our results show that, despite the small improvements in prediction accuracy, there were marked improvements in the bias estimates of genomic selection when subspecies of origin effects of alleles were accounted for. This finding is in accordance with a recent study in broiler chickens that showed that the incorporation of breed of origin alleles in crossbred genomic evaluations reduced bias in breeding value estimation (Duenk et al. 2019). These results show that consideration of ancestral origins in genomic prediction models can result in less biased, if not more accurate, multi-breed and multi-subspecies genomic evaluations.

By accounting for ancestral allele origins in our analysis, we have identified five subspecies-specific SNP that have an effect upon reproductive maturity score and occur quite commonly within our hybrid populations. The SNP with the largest posterior probability of inclusion was identified as being *B. indicus × B. taurus* in origin, BOVINEHD1200027774, which falls within the protein-coding region of the gene *FLT* (Fms Related Receptor Tyrosine Kinase 1). This gene has been associated with folliculogenesis in rats (Celik-Ozenci et al. 2003) litter size in sheep (Xu et al. 2018; Ghiasi and Abdollahi-Arpanahi 2021), and pre-eclampsia in women (Ashar-Patel et al. 2017). The SNP with the second largest posterior probability of inclusion was identified as being from *B. taurus* origins, BOVINEHD0200001401. This SNP falls within the protein-coding region of the gene *MY07B* (Myosin VIIB) which has been shown in human studies to be upregulated in the placental tissues of pre-eclampsia patients (Mohamad et al. 2020) and to be one of the predicted targets for one of the top ten most abundant microRNA’s in human ovaries (Xu et al. 2016). The third most significant SNP was identified again in *B. taurus* origins, BOVINEHD0300022359. This SNP is in the protein-coding region of the gene *WLS* (WNT Ligand Secretion Mediator). In mice, a study has shown that *WLS* knockout animals had a significant decrease in fertility due to reduced ovary sizes, decreased number of follicles and lower numbers of corpus luteum, which are all essential for female fertility (Chen et al. 2021). Finally, a *B. indicus* SNP, BOVINEHD0400006614, is in the protein-coding region of the gene *DGKβ* (Diacylglycerol kinase β). In the pituitary of rats, *DGKβ* is expressed in the dopamine receptors which is involved in the phosphoinositide cycle within the pituitary, which is involved with downstream signalling cascades involved in hormone action (Hozumi et al. 2010). One of the downstream hormones that is affected by this signalling pathway is luteinising hormone (Johnson et al. 1993; Hozumi et al. 2010), which is a hormone that has a strong regulatory function in oestrus in cattle (Stevenson and Pulley 2016). Interestingly, these five SNP fall within protein-coding regions of genes that are associated with pituitary or fertility in other species, but have not been previously associated with fertility in cattle (Celik-Ozenci et al. 2003; Hozumi et al. 2010; Ashar-Patel et al. 2017; Xu et al. 2018; Mohamad et al. 2020; Chen et al. 2021; Ghiasi and Abdollahi-Arpanahi 2021). These results show that, by accounting for the ancestral origin of SNP in hybrid populations, we were able to map novel, subspecies-specific QTL affecting RMS to genes that have a biological function in fertility in other species. In contrast, in other studies in beef cattle, a SNP in the region of the *PLAG1* gene was very significantly associated with early puberty (Fortes et al. 2013), but this SNP was not detected in our QTL association mapping results. However, after further investigation we observed that this SNP was almost fixed in the validation population, thus explaining why we were unable to detect it in our analysis.

Our method differs from many other multi-breed, allele breed of origin models in that (i) it uses a Bayesian approach, with a prior assumption of zero, very small or moderate effect of QTL, and (ii) no correlation between allele effects between subspecies is assumed. The latter seems appropriate for subspecies which diverged so long ago (Bradley et al. 1996; Hiendleder et al. 2008). It could be argued that the latter assumption may decrease accuracy of genomic predictions and precision of QTL mapping; however, all the evidence thus far points to different QTL segregating in *B. indicus* and *B. taurus* (Bolormaa et al. 2011). In cases where the same QTL segregates in both subspecies the QTL is clearly an introgression, usually from *B. taurus* into *B. indicus*, for example *PLAG1* (Fortes et al. 2013; Utsunomiya et al. 2017) and the polled mutation (Koufariotis et al. 2018). Note that our method does not preclude the effects of a SNP being correlated across subspecies, it just does not use this information. Our method could be extended to estimate correlations between pairs of marker effects across breeds.

It has been previously demonstrated that haplotypes may be used to detect regions of the genome that are either *B. indicus* or *B. taurus* in origin (Bolormaa et al. 2011; Koufariotis et al. 2018). Haplotypes can be constructed in a number of ways, such as using a fixed number of SNP per haplotype (Hayes et al. 2007; Villumsen et al. 2009), fixed chromosome lengths in cM (Boichard et al. 2012), fixed base-pair lengths (Hess et al. 2017) or by using linkage disequilibrium information to determine haplotypes of various lengths (Cuyabano et al. 2015). Furthermore, a number of studies have shown that optimising haplotype size is critical for the accuracy of genomic predictions and QTL mapping (Villumsen et al. 2009; Calus et al. 2009; Hess et al. 2017; Bian et al. 2021). Previous studies in Australian populations of *B. indicus* and *B. taurus* cattle have shown that fixed window haplotypes of 9–17 SNP (Bolormaa et al. 2011) or 250 kb (Koufariotis et al. 2018) were adequate at detecting subspecies differences in these populations. Our study also shows that use of fixed window haplotypes of 100 kb is optimal for tracing subspecies of origin effects through the genome of a hybrid population of tropically adapted cattle. The 100 kb window is smaller than the 250 kb proposed by Koufariotis et al. (2018) but slightly larger than the 9–17 SNP proposed by Bolormaa et al. (2011), which is equivalent to the 50 kb haplotype window size used in this study (Supplementary Table 1). Our results show that both the 50 kb and 250 kb haplotype window were able to trace subspecies of origin regions through the genome of this hybrid population, but the 100 kb haplotype window resulted in more accurate and less biased genomic predictions, whilst also being slightly more consistent with theoretical estimates of subspecies content. Studies in the literature have shown that optimisation of haplotype size is critical for obtaining the most accurate and unbiased genomic predictions (Villumsen et al. 2009; Calus et al. 2009; Hess et al. 2017; Bian et al. 2021). It is likely that longer haplotypes are persistent across closely related populations (Tang et al. 2006; Kling and Tillmar 2019), however in more distantly related populations, it is more likely that a recombination event will occur in long haplotypes over time (Villumsen et al. 2009). As such, smaller haplotype windows are more likely to persist across genetically distant populations (Hill and Weir 2011). It was beyond the scope of this paper to test the efficacy of different methods of defining haplotypes in this population to account for subspecies of origin effects. However, it may be beneficial to investigate the effect of defining haplotypes using other methods, such as linkage disequilibrium pruning, in a future study.

Haplotypes were only used to allocate SNP to a subspecies of origin and therefore genomic prediction was performed using SNP and not haplotypes. As haplotypes encompass a region of the genome containing neighbouring genetic markers, it is likely that haplotype alleles are in higher linkage disequilibrium with QTL than the single SNP alleles used in SNP genomic predictions (Zondervan and Cardon 2004). Linkage disequilibrium between SNP and QTL is essential for accurate genomic selection (Goddard 2009). Thus, if haplotypes increase the LD with QTL, it is hypothesised that the accuracy of genomic selection will be improved using haplotypes rather than single SNP (Hess et al. 2017). However, previous genomic predictions using haplotypes rather than SNP have shown mixed results in admixed populations (Hess et al. 2017; Araujo et al. 2021). A simulation study of a genetically diverse, admixed sheep population showed that there was no added benefit to using haplotype genomic prediction over SNP predictions in both prediction accuracy and bias (Araujo et al. 2021). In comparison, a study of an admixed dairy population, consisting of *B. taurus* breeds only, Hess et al. (2017) demonstrated that haplotype genomic predictions can result in improvements in the accuracy of genomic selection for a number of milk traits. It was noted, however, that the computation time of the haplotype genomic prediction analysis was significantly increased due to the increased number of covariates within the model (Hess et al. 2017). Particularly given the last point, with a view to implementing our predictions in routine genomic evaluations, we have elected to go with SNP based predictions.

One of the limitations to using haplotypes in genomic selection is the increased computing time required to phase haplotypes, define haplotype windows and to convert haplotypes to bi-allelic SNP format that can be used in existing genomic prediction pipelines (Teissier et al. 2020; Araujo et al. 2021). Haplotypes are more polymorphic than SNP as they are often multi-allelic (Meuwissen et al. 2014). In genetically diverse populations, such as *B. indicus* and *B. taurus* beef cattle populations, it is expected that there will be many unique haplotype alleles per loci. As such, many more rare haplotype alleles per loci may require further filtering before performing genomic predictions (Hess et al. 2017; Araujo et al. 2021). Hess et al. (2017) demonstrated the benefit of using a Bayesian analysis that allowed for unequal variant variances in haplotype genomic selection. As many Bayesian analyses allow variants to have no effect upon the trait of interest, rare haplotypes will have a small affect upon the trait and therefore, their effect will be shrunk towards zero (Gianola 2013). Hess et al. (2017) demonstrated that not filtering rare haplotype variants in Bayesian analyses had little impact upon the prediction accuracy of genomic predictions. However, it did result in improved computational times as there were fewer variants in the genomic prediction analyses. The BayesR framework used in our analysis allows variants to belong to one of four distributions: variants with no effect, variants with very small effect, variants with small effect and variants with moderate to large effect. As this framework allows rare variants to have no effect, we did not use MAF filters to filter out rare haplotypes in this study. It may be beneficial to investigate improvements in computation times using this analysis after rare variants have been filtered, in future studies.

The *B. taurus* reference population used in this study consisted of 313 animals from 5 pure *B. taurus* breeds, Angus (*n* = 100), Hereford (*n* = 43), Limousin (*n* = 62), Shorthorn (*n* = 95) and Charolais (*n* = 13) whereas the *B. indicus* reference population consisted of 868 animals from a single breed, Brahman. Within breeds it is expected that animals will share more haplotypes as there is increased probability that they have recent ancestors in common (Hill and Weir 2011). In the multi-breed *B. taurus* reference population, frequencies of haplotypes may vary between breeds which may potentially result in a lot of haplotypes with low frequency at each loci in this population. In comparison, the *B. indicus* population has a large number of animals from only a single breed, which may result in fewer haplotype alleles with higher frequencies, as it is more likely that these animals will share recent common ancestry and thus share haplotypes in common. In this study, reference population haplotype frequency is used to assign validation population haplotype subspecies of origin. If a haplotype occurs in both subspecies, Bi and Bt, it will be more likely to be assigned to the subspecies with the highest frequency haplotype in the reference population. Therefore, the single breed *B. indicus* population may be biasing the assignment of some haplotypes towards a *B. indicus* subspecies of origin. Also, as previously stated, the 250 kb haplotype window may be too long to accurately differentiate between these genetically divergent subspecies and, coupled with this potential *B. indicus* subspecies allocation bias, more of the 250 kb haplotypes may have been allocated to the *B. indicus* subspecies of origin. Particularly in comparison to the 100 kb and 50 kb haplotype windows and in reference to the theoretical breed origins. In future studies, it would be beneficial to use larger reference populations, with a multi-breed *B. indicus* reference population, to reduce any potential impact of population structure when assigning haplotype subspecies of origin.

There were three main limitations to our study, the absence of mixed breed contemporary groups, the lack of purebred *B. taurus* animals in our validation population, and the absence of a purebred *B. indicus* breed in our reference population. Accurate multi-breed genomic selection requires direct comparisons between breeds. Our validation dataset consisted of single breed contemporary groups, thus there were no direct head-to-head comparisons between each breed to enable accurate comparison of breed effects. Secondly, as there were no purebred *B. taurus* animals in our validation population, all *B. taurus* haplotypes in our validation dataset originated from the *B. indicus × B. taurus* stabilised composite breeds. More research is required to determine if the inclusion of purebred *B. taurus* animals in our validation dataset would further improve the accuracy of our subspecies-specific BayesR analyses. Finally, the Australian Brahman population has a known proportion of *B. taurus* introgression (~10%) (Bolormaa et al. 2011; Koufariotis et al. 2018). As Brahmans are not a purebred *B. indicus* breed, there may be error in the subspecies of origin assignment of some *B. indicus* haplotypes in this study. The impact of this incorrect assignment is likely to be minimal, as our results have shown that the subspecies of origin haplotype assignment was able to assign 10% of the Brahman genome to *B. taurus* origins. However, future studies using a purebred *B. indicus* breed in the reference population will be required to test the efficacy of this method.

In this paper, we have developed a method to simultaneously map subspecies-specific effects in hybrid populations, in order to better understand the underlying genomic architecture of genetically diverse populations. It is essential to identify the appropriate haplotype window size to use in each population to optimise accuracy of both QTL mapping and genomic evaluations. However, our method demonstrated that, in the absence of pedigree information, marker haplotypes can be used to accurately assign ancestral subspecies of origin to genomes. When used in conjunction with our BayesR analysis, we were able to identify novel QTL that have not previously been identified in cattle, but were closely linked to genes biologically involved with fertility in other species.

### Supplementary information

Supplementary Figure 1

Supplementary Table 1

## Data Availability

Summary statistics of the Bayesian analyses described in this study are publicly available in Dryad https://datadryad.org/stash/share/07ONnitFJ3EZOVOG7nnAC5yeeFbpJ3HPWWTtZfgSb0Q. Scripts used to produce haplotypes, calculate subspecies of origin and create X-matrices will be publicly available on GitHub https://github.com/cwarburton85/Subspecies_Xmatrix.
