# Supplementary material for: Concurrently mapping quantitative trait loci associations from multiple subspecies within hybrid populations
Source: Heredity (Edinb). 2023 Oct 6;131(5-6):350–60. doi: 10.1038/s41437-023-00651-4 (PMC10673866; doi:10.1038/s41437-023-00651-4)
Supplement: Supplementary file 1 — Supplementary Figure 1 [file 41437_2023_651_MOESM1_ESM.docx]

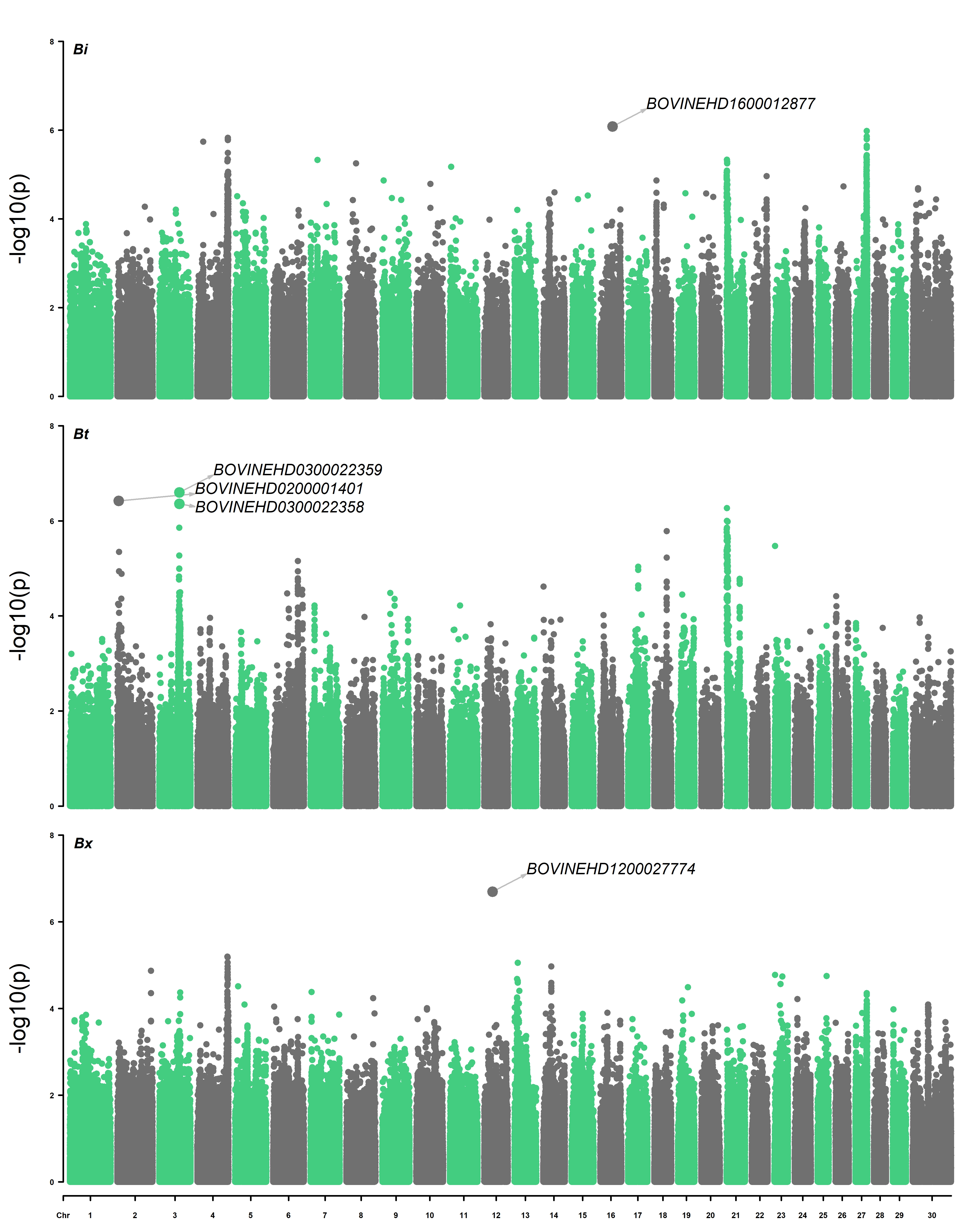


**Supplementary Figure 1 – Genome wide association study (log10(p)) of subspecies-specific SNP effects, Bos indicus (Bi), Bos taurus (Bt) and Bos indicus x Bos taurus (Bx), using the 100kb haplotype defined subspecies SNP.**
