# Supplementary material for: Concurrently mapping quantitative trait loci associations from multiple subspecies within hybrid populations
Source: Heredity (Edinb). 2023 Oct 6;131(5-6):350–60. doi: 10.1038/s41437-023-00651-4 (PMC10673866; doi:10.1038/s41437-023-00651-4)
Supplement: Supplementary file 2 — Supplementary Table 1 [file 41437_2023_651_MOESM2_ESM.docx]

**Supplementary Table 1 – Average number of SNP that fall within a haplotype window for each chromosome and each fixed haplotype length for the Smart Futures Heifers.**

| **Chromosome** | **50kb** | **100kb** | **250kb** |
| --- | --- | --- | --- |
| **1** | 13.91 | 27.77 | 69.34 |
| **2** | 13.91 | 27.75 | 69.26 |
| **3** | 13.88 | 27.69 | 69.10 |
| **4** | 13.72 | 27.35 | 68.29 |
| **5** | 13.60 | 27.07 | 67.43 |
| **6** | 14.24 | 28.42 | 70.85 |
| **7** | 14.18 | 28.14 | 70.07 |
| **8** | 14.27 | 28.30 | 70.28 |
| **9** | 13.91 | 27.79 | 69.45 |
| **10** | 14.00 | 27.76 | 69.06 |
| **11** | 14.14 | 28.22 | 70.42 |
| **12** | 14.09 | 27.76 | 68.00 |
| **13** | 13.34 | 26.50 | 65.97 |
| **14** | 14.29 | 28.45 | 70.53 |
| **15** | 13.60 | 26.92 | 67.08 |
| **16** | 14.09 | 28.09 | 69.97 |
| **17** | 14.24 | 28.26 | 70.12 |
| **18** | 13.90 | 27.57 | 68.60 |
| **19** | 13.86 | 27.60 | 68.74 |
| **20** | 14.14 | 28.26 | 70.64 |
| **21** | 13.93 | 27.68 | 69.06 |
| **22** | 13.99 | 27.91 | 69.60 |
| **23** | 13.81 | 27.37 | 67.84 |
| **24** | 14.23 | 28.30 | 70.46 |
| **25** | 14.10 | 28.11 | 70.10 |
| **26** | 13.92 | 27.79 | 69.40 |
| **27** | 14.09 | 27.86 | 69.03 |
| **28** | 13.24 | 26.42 | 66.11 |
| **29** | 13.56 | 26.95 | 67.11 |
| **30** | 12.77 | 25.08 | 61.13 |
| **Average** | **13.90** | **27.64** | **68.78** |
